# Supplementary material for: Vitamin D Modified DSS-Induced Colitis in Mice via STING Signaling Pathway
Source: Biology (Basel). 2025 Jun 18;14(6):715. doi: 10.3390/biology14060715 (PMC12190092; doi:10.3390/biology14060715)
Supplement: Supplementary file 1 [file biology-14-00715-s001.zip › Supplementary Materials S1: 16s-report of gut microbiota/Groups/diff_analysis/TukeyHSD/Tukey_GBM.pdf]

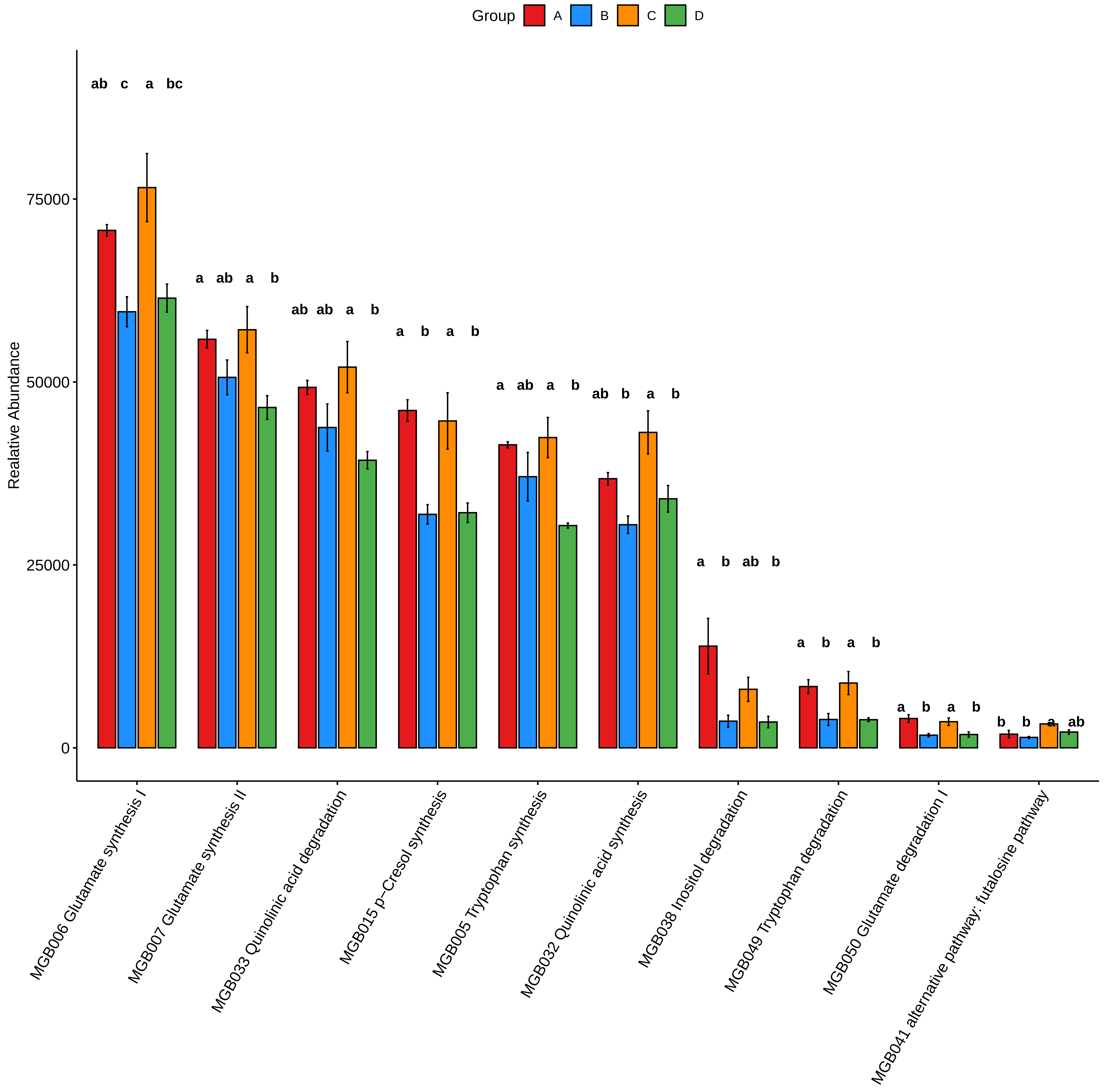
Group
A
B
C
D
Realative Abundance
75000
50000
25000
0
ab c a bc
a ab a b
ab ab a b
a b a b
a ab a b
ab b a b
a b ab b
a b a b
a b a b
b b a ab
MGB006 Glutamate synthesis I
MGB007 Glutamate synthesis II
MGB033 Quinolinic acid degradation
MGB015 p−Cresol synthesis
MGB005 Tryptophan synthesis
MGB032 Quinolinic acid synthesis
MGB038 Inositol degradation
MGB049 Tryptophan degradation
MGB050 Glutamate degradation I
MGB041 alternative pathway: futalosine pathway
